# Computer assisted total knee arthroplasty for osteoarthritis to secondary to congenital dislocation of the patella: A case report

**DOI:** 10.1016/j.ijscr.2018.11.010

**Published:** 2018-11-13

**Authors:** Kwangkyoun Kim

**Affiliations:** Konyang University, College of Medicine, Department of Orthopaedic Surgery, Konyang University, 158 Gwanjeodong-ro, Seo-gu, Daejeon, Republic of Korea

**Keywords:** Knee, Osteoarthritis, Congenital dislocation of patella, Total knee arthroplasty, Computer assisted surgery

## Abstract

**Introduction:**

Late presentation of congenital patella dislocation (CPD) with advanced osteoarthritis is very rare. Total knee arthroplasty (TKA) provides a valid treatment option for adults with CPD who have absence of the femoral sulcus and associated osteoarthritis. TKA for knee with CPD demand more precise technique because TKA correct the unusual pathologies for primary osteoarthritis, eg, tibial external rotation, absence of femoral groove, patella hypoplasia, and realignment of extensor mechanism.

**Case presentation:**

This case is a CPD with advanced osteoarthritis of left knee in a seventy-two-year old woman. We used a computer assisted navigation for a restoration of alignment, extension and flexion gap balancing, and mediolateral symmetry in the TKA, and operated it successfully.

**Conclusion:**

TKA is a useful procedure for osteoarthritis of the knee in association with CPD. In hard case of osteoarthritis with CPD, computer assisted navigation is a useful tool for the TKA.

## Highlights

• Late presentation of congenital patella dislocation (CPD) with advanced osteoarthritis is very very rare.
• It is very difficult to perform a total knee arthroplasty (TKA) with conventional method.
• This is the first case report of with advanced osteoarthritis operated with computer navigated TKA.
• A CPD with osteoarthritis treated TKA successfully and discussed points to note during opertaion using computer navigation system.

## 1 Introduction

Congenital patella dislocation (CPD) comprises a pathological condition of permanent lateral dislocation of this bone. It is impossible to reduce it through manual maneuvers. It is generally diagnosed at birth. These infants present with genu valgum and contracture of the flexed knee, in association with external rotation of the tibia. When these deformities are not present, this pathological condition may not be diagnosed until adulthood is reached [1]. With early diagnosis and surgical correction, severe sequelae of CPD, osteoarthritis is very rare.

Total knee arthroplasty (TKA) provides a valid treatment option for adults with CPD who have associated osteoarthritis. Most of cases reports about TKA for CPD discuss about realignment of extensor mechanism [[2], [3], [4]]. However, for achieving the goals of TKA, it is very important to restore a flexion – extension gap balance and mediolateral ligament symmetry. More precise technical considerations are demanded in CPD with osteoarthritis because of lack of anatomical landmarks for alignment for implants position, fixed valgus alignment, and tibial external rotation.

The author reported a neglected CPD with osteoarthritis treated TKA and discussed the precise technique about making flexion–extension gap balance and mediolateral ligament symmetry using computer assisted navigation system.

## 2 A case presentation

**Fig. 1:**
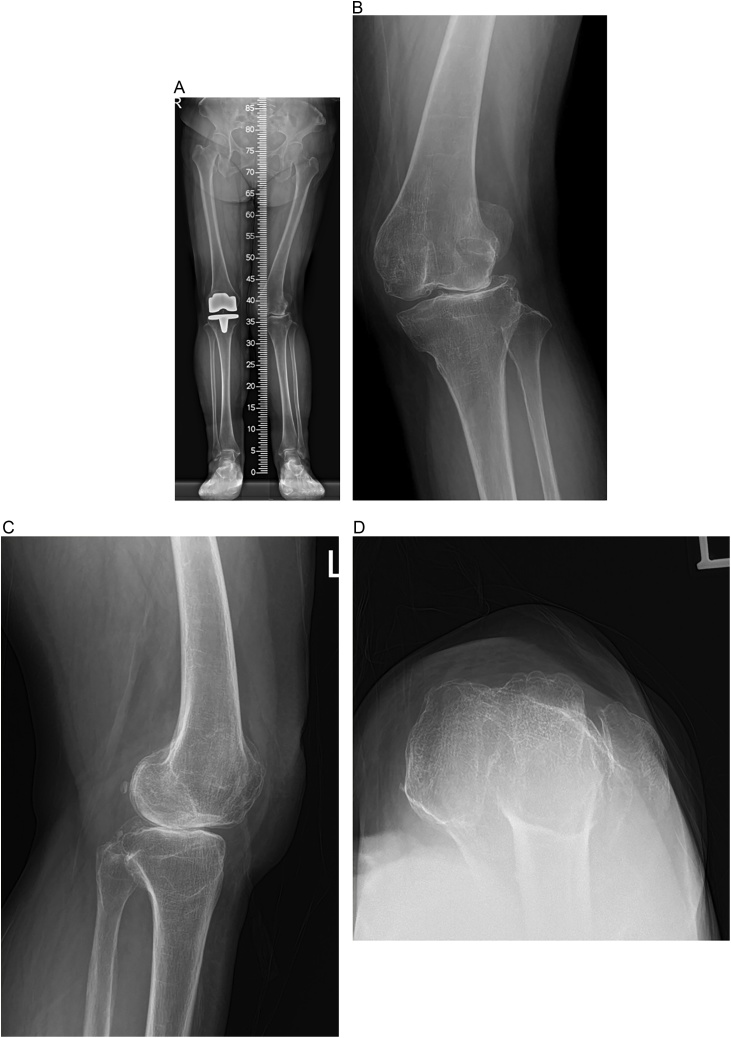
Pre-operative radiograph (A) On low extremity standing scanogram, left knee shows severe valgus alignment. (B) on anteroposterior weight-bearing radiograph, left knee shows severe valgus osteoarthritis with lateral dislocation of the patella (C) on lateral radiography, patella was not seen. (D) On skyline view, patella is dislocated laterally.

A 72-year-old woman had a 7-year history of left knee pain. She was a housewife and her body mass index was 22 kg/m^2^. She had no neurologic condition. However, she frequently fell down because of knee instability and pain. She was treated for distal ulnar and radius fracture two years ago and scaphoid fracture six months ago. She had been diagnosed with bilateral congenital patella dislocation with osteoarthritis. She underwent TKA for the right knee 10 years ago. She delayed TKA for the left knee because she was not satisfied with the TKA for the right knee which had subluxated patella and limited active range of motion from 10° to 90° with extension lag. The left knee pain had gradually worsened over the last three years. She only walked with two crunches or frame since worsening instability and pain during gait of left knee. On examination, there was tenderness on the medial aspect of joint line which was aggravated by motion. Left knee revealed quadriceps atrophy. Quadriceps strength was rated as 4 on a muscle testing scale of 0 to 5. Patella was palpable at the lateral side of the femoral condyle. It had no mobility during flexion or extension. The tibia showed external rotation. The active range of motion was 20° to 135° and passive range of motion was measured 0° to 135°. Roentgenograms showed severe valgus deformity and severe osteoarthritis with patella dislocation of the left knee along with complete loss of lateral compartment joint space (Fig. 1).

**Fig. 2:**
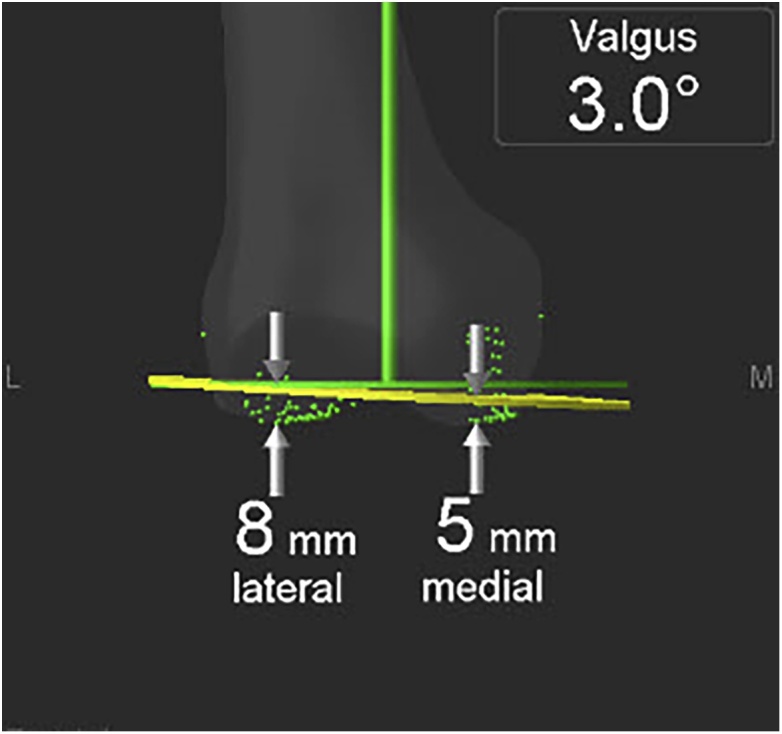
(A) Distal femur was resected with 3° valgus to the right angle to the mechanical axial on the coronal plane (B) graphic shows asymmetry of extension gap after bone resection and ligament release.

Midline skin incision and medial parapatellar arthrotomy was used. Vastus medialis muscle was found to be stretched over the anterior aspect of the distal femur. Quadriceps showed atrophy. Patella was hypoplastic while femoral grove for patella was absent (Fig. 2).

**Fig. 3:**
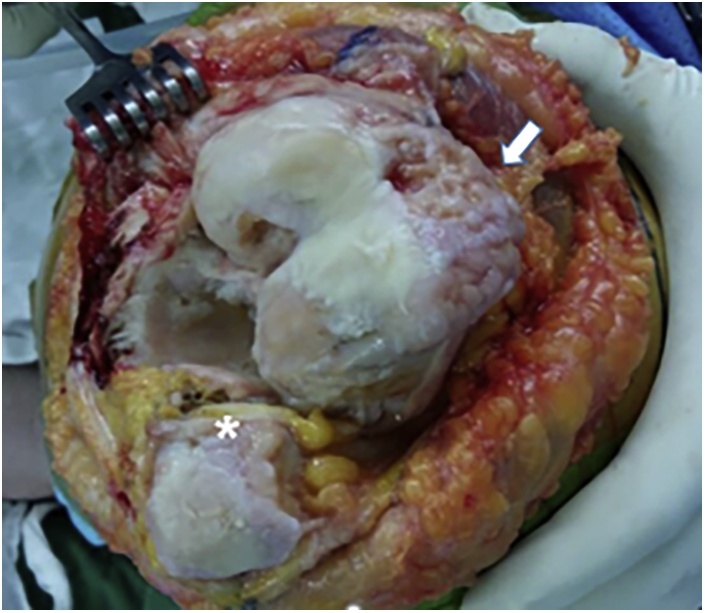
White arrow shows absence of distal femoral groove, and an asterisk shows patella hypoplasia.

The patient underwent a left TKA with computer navigation using a Stryker 4.0 system (Stryker Co., Allendale, NJ, USA). Resection of the proximal tibia was performed first. This cut was verified to confirm that the desired resection was carried out as this plane could be the reference plane for further femoral cuts. To reduce extensive lateral soft tissue release for gap balancing and relocation of the patella, authors cut distal femur with valgus 3° to the right angle to mechanical axis (Fig. 3). After cutting the bone, author released lateral ligament structure for mediolateral symmetry of the extension gap.

**Fig. 4:**
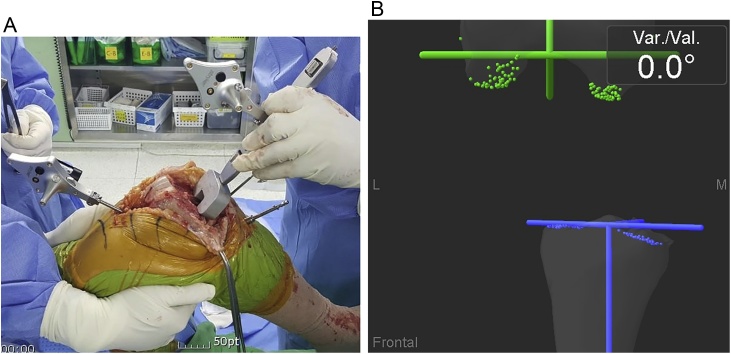
(A) A custom spacer with a navigation tracker attached was placed the resected tibia with the knee in 90° flexion with maintaining the tension by manual traction. (B) parallel line to the proximal tibial resection plane was displayed on the monitor, and was marked on the cut surface of distal femur.

Author could not use any bony land mark on the femur for femoral component rotation because femoral condyle was hypoplastic with no femoral groove, epicondyles obscure, and posterior lateral condylar hypoplasia. Author used tibial cutting surface as the landmark for rotational alignment for femoral component. With the knee flexed to 90° and maintaining the tension by traction, spacer blocks of extension gap thickness with computer trackers were placed on the tibial cutting surface (Fig. 4A). When parallel line to the proximal tibial resection plane was displayed on the monitor, the surgeon marked a line on the cut surface of distal femur (Fig. 4B).

**Fig. 5:**
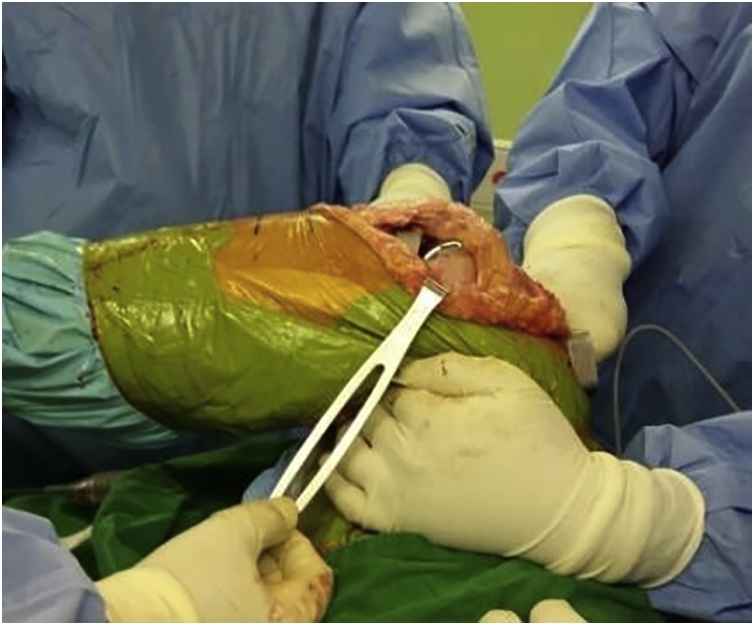
After the extensor realignment, patella tracked centrally in the patellofemoral groove located centrally throughout the range of motion, and the hip was flexed 90° and passive knee flexion against gravity was 90°.

For proximal realignment of patella, extensive lateral retinaculum, lateral collateral ligament release, and quadriceps tendon lengthening were performed using Vulpius technique. Additionally, the medial aspect of capsule and vastus medialis obliquus were brought over the top of the inverted V-shaped pedicle and secured in position with a double row of sutures.^3^ After the repair was complete, the patella tracked centrally in the patellofemoral groove, the hip was flexed 90°, and passive knee flexion against gravity was 90° (Fig. 5). Patella was not resurfaced because its thickness was 12 mm.

**Fig. 6:**
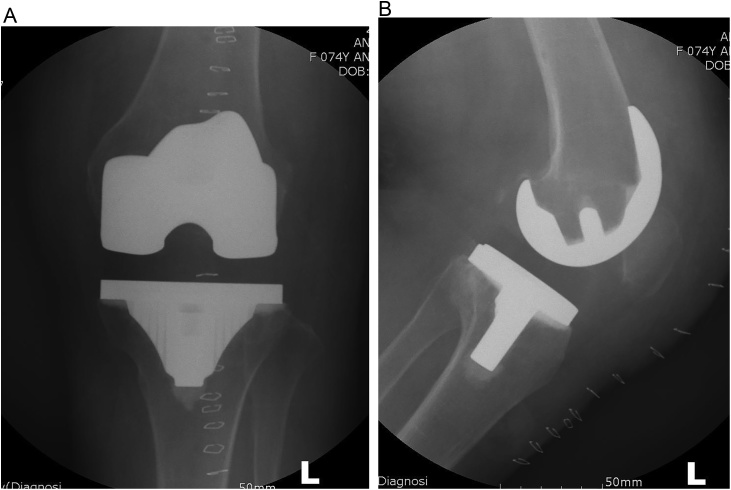
Immediate post-operative radiograph (A) Anteroposterior fluoroscopy showed well positioned implant and mediolateral symmetry (B) lateral fluoroscopy showed a patella on the front of femur that had not seen on the preoperative lateral radiography.

**Fig. 7:**
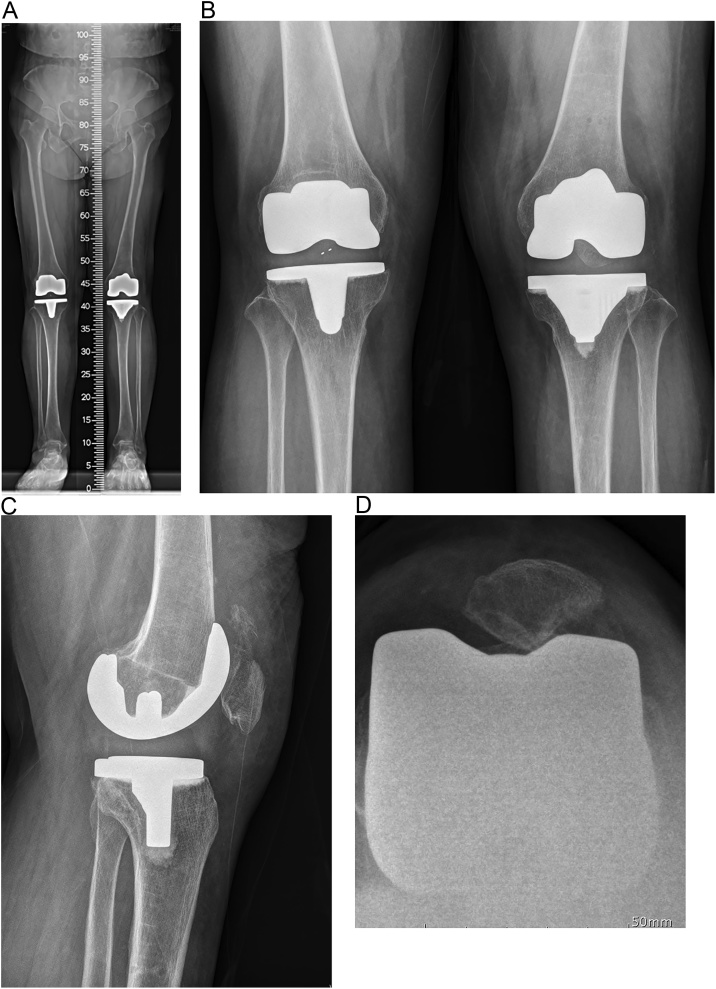
At post-operative 1 year (A) On low extremity standing scanogram, left lower extremity shows a restoration of alignment with 3° residual valgus, but it is within an acceptable range. (B) weight bearing AP view, patella was located on front of femur (C) at post-operative 1 year, patella was seen well on the weight bearing lateral view. (D) on skyline view, patella was placed in the groove of the femoral component with minimal lateral displacement.

Immediate post-operatively, reduction of patella was confirmed with fluoroscopy (Fig. 6). The knee was immobilized in full extension in functional brace for 1 week. Exercise and walking training with a walker were then started. Early range of motion and quadriceps strengthening exercise were encouraged but flexion was limited to 90 ° for 3 weeks. After post-operative 4 weeks, the patient was encouraged to exercise without limitation of range of motion. At final follow up (1 year postoperatively), her left knee was pain-free with an active range of motion of 0° to 120° without extension lag. There was no evidence of patella maltracking or instability on physical examination. However, radiography showed minimal patellar lateral displacement (Fig. 7).

## 3 Discussion

Osteoarthritis with CPD is rare. There are few reports for TKA with this condition. To achieve good results, surgeons demand more precise technical consideration because landmarks are lacking for alignment of implants position and fixed valgus alignment with shorten extensor mechanism and external rotated tibia.

Many studies have demonstrated that computer-assisted navigation system can help ensure accurate evaluation of the mechanical axis in frontal and sagittal planes by kinematic registration of the hip, knee, and ankle centers. It can also adjust bone cut and soft tissue balance delicately and easily [[7], [8], [9], [10], [11]].

There are two technical points to consider in computer assisted TKA in CPD. First, tibia cut first and gap-based navigation technique is useful for femoral component rotation because of obscure anatomical landmarks in CPD patients. Second, surgeons should consider tibial external torsion of a CPD patient and try to avoid transcortical pin misplacement, especially for an obese patient. Previously, an iatrogenic tibial stress fracture after computer navigated TKA has been reported. The stress fracture occurred at one of pinhole sites used for placing tibial trackers [12].

Osteoarthritis with CPD is usually valgus alignment. It needs release of lateral collateral ligament and/or the popliteus tendon. An excessive valgus release may lead to posterolateral instability in flexion. According to Pradhan et al. [3], if an excessive release is necessary, a constrained type prosthesis should be considered first. Traditionally, TKA has been considered successful when a neutral mechanical hip-knee-ankle axis within 3° is achieved. Recently, it has been reported that slight under-correction following TKA for a valgus knee does not affect clinical outcomes or implant survival [13,14]. In our case, authors placed femoral component with valgus 3° to the right angle to mechanical axis to reduce extensive soft tissue release for gap balancing, mediolateral symmetry, and relocation of the patella. Computer assisted TKA is able to control the adjustment of the bone cut and soft tissue balance with aid of digital system delicately and easily [7,8].

In our case, patella was reduced centrally. There is no consensus about relocating extensor mechanism. Marmor [5] and Pradhan et al. [6] reported that they successfully treated bilateral TKA without attempting to correct dislocated patella and extensor mechanism. However, these reports did not present functional results at long-term follow up. Other authors have corrected the extensor mechanism and relocated the patella using lateral release and/or vastus medialis advancement with excellent results [[2], [3], [4]]. Relocate extensor mechanism has potential for regaining active extension and losing the pre-operative extension lag. However, it also has potential for reducing knee flexion. No relocating of the dislocated patella has potential for remaining extension lag. Thus, surgeons need to know what patients wish to obtain with regard to whether the patella is or is not reduced, and if necessary, how to realign the extensor mechanism.

## 4 Conclusion

TKA is a useful procedure for osteoarthritis of the knee in association with CPD. In difficult case of osteoarthritis with CPD, computer assisted navigation is a useful tool for restoring alignment, extension and flexion gap balancing, and mediolateral symmetry of the TKA.

"Written informed consent was obtained from the patient for publication of this case report and accompanying images. A copy of the written consent is available for review by the Editor-in-Chief of this journal on request”.

## Conflicts of interest

None.

## Funding

None.

## Ethical approval

The patient provided consent for data concerning this case to be submitted for publication and approved by the internal review board of our institution (KYUH 2018-03-007).

## Consent

Written informed consent was obtained from the patient for publication of this case report and accompanying images. I attached a informed consent.

## Author contribution

Kwangkyoun Kim did all of study concept or design, data collection, data analysis or interpretation, writing the paper.

## Registration of research studies

This case report is not a research involving human participants.

## Guarantor

Kwangkyoun is the guarantor responsibility for the work and/or the conduct of the study, had access to the data, and controlled the decision to publish.

## Provenance and peer review

Not commissioned, externally peer reviewed.
